# Impact of Lesion Load Thresholds on Alberta Stroke Program Early Computed Tomographic Score in Diffusion-Weighted Imaging

**DOI:** 10.3389/fneur.2018.00273

**Published:** 2018-04-23

**Authors:** Julian Schröder, Bastian Cheng, Caroline Malherbe, Martin Ebinger, Martin Köhrmann, Ona Wu, Dong-Wha Kang, David S. Liebeskind, Thomas Tourdias, Oliver C. Singer, Bruce Campbell, Marie Luby, Steven Warach, Jens Fiehler, André Kemmling, Jochen B. Fiebach, Christian Gerloff, Götz Thomalla

**Affiliations:** ^1^Klinik und Poliklinik für Neurologie, Kopf- und Neurozentrum, Universitätsklinikum Hamburg-Eppendorf, Hamburg, Germany; ^2^Institut für Computational Neuroscience, Universitätsklinikum Hamburg-Eppendorf, Hamburg, Germany; ^3^Centrum für Schlaganfallforschung Berlin, Charité – Universitätsmedizin Berlin, Berlin, Germany; ^4^Klinik für Neurologie, Charité – Universitätsmedizin Berlin, Berlin, Germany; ^5^Klinik für Neurologie, Universität Erlangen-Nürnberg, Erlangen, Germany; ^6^Athinoula A. Martinos Center for Biomedical Imaging, Department of Radiology, Massachusetts General Hospital, Harvard Medical School, Boston, MA, United States; ^7^Department of Neurology, Asan Medical Center, University of Ulsan College of Medicine, Seoul, South Korea; ^8^Neurovascular Imaging Research Core, Department of Neurology, University of California, Los Angeles, Los Angeles, CA, United States; ^9^Service de Neuroimagerie Diagnostique de Thérapeutique, Centre Hospitalier Universitaire de Bordeaux, Université de Bordeaux, Bordeaux, France; ^10^Klinik für Neurologie, Universitätsklinikum Frankfurt, Frankfurt, Germany; ^11^Department of Medicine and Neurology, Melbourne Brain Centre at the Royal Melbourne Hospital, University of Melbourne, Melbourne, VIC, Australia; ^12^National Institute of Neurological Disorders and Stroke (NINDS), National Institutes of Health (NIH), Bethesda, MD, United States; ^13^Department of Neurology, Dell Medical School, University of Texas at Austin, Austin, TX, United States; ^14^Klinik und Poliklinik für Neuroradiologische Diagnostik und Intervention, Universitätsklinikum Hamburg-Eppendorf, Hamburg, Germany; ^15^Institut für Neuroradiologie, Universitätsklinikum Schleswig-Holstein, Lübeck, Germany

**Keywords:** stroke, acute stroke treatment, computed tomography, magnetic resonance imaging, Alberta stroke program early computed tomography score

## Abstract

**Background and aims:**

Assessment of ischemic lesions on computed tomography or MRI diffusion-weighted imaging (DWI) using the Alberta Stroke Program Early Computed Tomography Score (ASPECTS) is widely used to guide acute stroke treatment. However, it has never been defined how many voxels need to be affected to label a DWI-ASPECTS region ischemic. We aimed to assess the effect of various lesion load thresholds on DWI-ASPECTS and compare this automated analysis with visual rating.

**Materials and methods:**

We analyzed overlap of individual DWI lesions of 315 patients from the previously published predictive value of fluid-attenuated inversion recovery study with a probabilistic ASPECTS template derived from 221 CT images. We applied multiple lesion load thresholds per DWI-ASPECTS region (>0, >1, >10, and >20% in each DWI-ASPECTS region) to compute DWI-ASPECTS for each patient and compared the results to visual reading by an experienced stroke neurologist.

**Results:**

By visual rating, median ASPECTS was 9, 84 patients had a DWI-ASPECTS score ≤7. Mean DWI lesion volume was 22.1 (±35) ml. In contrast, by use of >0, >1-, >10-, and >20%-thresholds, median DWI-ASPECTS was 1, 5, 8, and 10; 97.1% (306), 72.7% (229), 41% (129), and 25.7% (81) had DWI-ASPECTS ≤7, respectively. Overall agreement between automated assessment and visual rating was low for every threshold used (>0%: κ_w_ = 0.020 1%: κ_w_ = 0.151; 10%: κ_w_ = 0.386; 20% κ_w_ = 0.381). Agreement for dichotomized DWI-ASPECTS ranged from fair to substantial (≤7: >10% κ = 0.48; >20% κ = 0.45; ≤5: >10% κ = 0.528; and >20% κ = 0.695).

**Conclusion:**

Overall agreement between automated and the standard used visual scoring is low regardless of the lesion load threshold used. However, dichotomized scoring achieved more comparable results. Varying lesion load thresholds had a critical impact on patient selection by ASPECTS. Of note, the relatively low lesion volume and lack of patients with large artery occlusion in our cohort may limit generalizability of these findings.

## Introduction and Aims

The Alberta Stroke Program Early Computed Tomography Score (ASPECTS) is widely used in clinical practice to assess the extent of early ischemic changes on brain imaging for acute stroke treatment. Introduced for standardized evaluation of non-contrast computed tomography (CT) the ASPECTS template consists of 10 regions distributed throughout the middle cerebral artery (MCA) territory. For each affected region, the overall score is reduced by 1 from a score of 10 that indicates a normal scan. Originally, a threshold of ASPECTS ≤7 was proposed to identify patients at high risk for intracerebral hemorrhage and poor clinical outcome (1). The template was also applied to diffusion-weighted MRI (2, 3) and perfusion imaging on both CT and MRI (4).

Three of the recently published trials demonstrating efficacy of mechanical thrombectomy for treatment of acute stroke used ASPECTS to exclude patients presumed to have a large ischemic core. The ESCAPE trial excluded patients with ASPECTS <6 (5), the REVASCAT study used a cut-off of ASPECTS <7 (5) to exclude patients based on CT, and of ASPECTS <6 if diffusion-weighted imaging (DWI) was applied. SWIFT-PRIME used NCCT- or DWI-ASPECTS ≤5 as an exclusion threshold (6). Furthermore, interventional treatment was shown to be particularly effective in patients with ASPECTS 8–10 (5, 7). Additionally, clear benefit of endovascular stroke treatment was not observed in patients with a low ASPECTS value between 0 and 5 (8). Based on these results, and inclusion of an ASPECTS ≥6 threshold in the AHA guideline for endovascular thrombectomy (9), expansion of the use of ASPECTS in clinical practice is likely.

However, there are limitations to ASPECTS. Individual ASPECTS regions are not equally weighed, and correlation with stroke lesion volume is low in subcortical regions (10, 11). Latter limitations stem from definition and distribution of ASPECTS regions as well as from the fact that the exact dimensions of each region are rather vaguely defined. Moreover, although originally designed for use with non-contrast CT, ASPECTS is increasingly used to evaluate DWI (2, 3) which poses another challenge. DWI is highly sensitive in the detection of small, even punctuate, ischemic lesions (12). However, there is no guidance as to how many voxels on DWI must be lesioned to classify the region as being affected. As yet, there is no data on how visual rating of ASPECTS on DWI relates to the proportion of individual regions being affected.

In this study, we aimed to assess how different thresholds in quantitative evaluation of DWI-ASPECTS affect the overall score, and how quantitative analysis of DWI-ASPECTS compares to visual rating. For this purpose, we compared visual DWI-ASPECTS rating to different automated approaches based on the overlap of individual DWI lesions with a probabilistic ASPECTS template.

## Methods

We analyzed data from the predictive value of fluid-attenuated inversion recovery (PRE-FLAIR) study database. PRE-FLAIR was a multicenter retrospective study of patients with acute ischemic stroke who underwent multiparametric MRI within 12 h of symptom onset (13). NCCT was not performed within PRE-FLAIR, thus a comparison to NCCT ASPECTS was not feasible with this dataset.

Predictive value of fluid-attenuated inversion recovery was conducted by an international consortium of researchers within the Stroke Imaging Repository (STIR) and Virtual International Stroke Trials Archive (VISTA) Imaging research groups. PRE-FLAIR included individual datasets from eight participating stroke centers and two studies (13). The study was approved by the local ethics committees at all centers. Either written or verbal informed consent was obtained for all patients, as required by local legislation. PRE-FLAIR was registered with ClinicalTrials.gov, number NCT01021319 (13).

Demographic data, severity of neurological deficit on admission as assessed by National Institutes of Health Stroke Scale (NIHSS) were collected by individual centers. DWI-ASPECTS was rated by a neurologist with 7 years of experience in stroke imaging research and clinical application.

For the present analysis, we excluded patients with anterior or posterior cerebral artery infarction and bilateral ischemic lesions. Thus, we included only patients with unilateral MCA infarction. Furthermore, patients with insufficient image quality for processing and quantitative analysis were excluded.

A population-based probabilistic ASPECTS atlas was created based on 221 normal non-enhanced CT scans. All CT exams were performed according to the department’s standard protocol on a iCT 256™ scanner (Philips Healthcare, Best, The Netherlands): collimation 64 × 0.625, pitch 0.297, rotation time 0.4 s, FOV 270 mm, tube voltage 120 kV, tube current 300 mA, 4.0 mm slice reconstruction. ASPECTS regions (C, caudate; L, lentiform; IC, internal capsule; I, insular ribbon; M1, anterior MCA cortex, M2, MCA cortex lateral to insular ribbon, M3, posterior MCA cortex, M4, M5, and M6 are anterior, lateral, and posterior MCA territories immediately superior to M1, M2, and M3) were manually segmented on CT images by two raters using all slices covering the entire MCA territory (Analyze 11.0, Analyzedirect) (14). Binary ASPECTS maps based on each exam were then affine registered to standard Montreal Neurologic Institute (MNI) space with 12 degrees of freedom (FLIRT 5.5, FMRIB linear image registration tool) followed by non-linear refinement using a custom CT reference image (FNIRT 1.0, FMRIB non-linear image registration tool) (15).

Based on the individual binary ASPECTS region maps a probabilistic ASPECTS map was calculated. Each voxel within the ASPECTS map is thus characterized by its probability of belonging to any of the 10 ASPECTS regions. Please refer to the Datasheet S1 in Supplementary Material for further details.

Individual DWI lesions for each patient were segmented and lesion volumes calculated by a semiautomatic thresholding approach using an in-house developed software tool (AnToNIa) (16), as described previously (13). DWI lesions were manually surrounded with a generous safety margin at each affected slice. Intensity thresholding was applied to refine the defined lesion area. We retained all voxels that were part of the defined lesion area with a signal intensity exceeding the mean signal intensity of the unaffected hemisphere by more than two SDs and rejected all others.

The individual DWI maps and respective lesion masks were registered to MNI brain atlas space. All masks of individual DWI lesions were binarized (voxels with infarction had a value of 1, all others 0).

The overlap of each individual DWI lesion mask with each single ASPECTS region of the probabilistic template and the respective overlap volumes were calculated using imaging tools from the Functional MRI of the Brain Software Library (FMRIB Software Library; http://www.fmrib.ox.ac.uk/fsl).

By use of a probabilistic template, each voxel of the DWI lesion overlapping with an ASPECTS region was corrected for its probability of belonging to that respective region. Thus the resulting volume (of DWI lesion within an ASPECTS region) equals the overlap of a binarized lesion and template multiplied with the probability for the included voxels of belonging to any of the ASPECTS regions. Please refer to the Datasheet S1 in Supplementary Material for further details.

Based on the probabilistic volume of each ASPECTS region we calculated the percentage of lesioned tissue volume in each region, the relative lesion load (RLL) per ASPECTS region.

Therefore, RLL represents the share of infarction within a probabilistic ASPECTS region. For regions containing only healthy tissue the RLL would thus be 0%, for regions completely involved in the ischemic lesion RLL would be 100%.

Then multiple RLL thresholds (>0, >1, >10, and >20% of the respective ASPECTS region) were applied to define whether a region was considered affected. Overall scores were then calculated based on these results.

Median and interquartile range for all ASPECTS values and the agreement κ of visual ASPECTS with the automated assessment were computed. For overall scores weighted κ (κ_w_) with linear weights for each ASPECTS level was used to account for the ordinal nature of the scale. The Mann–Whitney-*U*-test was used for comparison of means. For correlation analysis, Spearman’s rank correlation coefficient was calculated. Statistical analysis was performed using SPSS 23, weighted κ was calculated with STATA/SE 14.1.

## Results

Of the 496 patients with MCA infarction in the PRE-FLAIR database, 181 had to be excluded due to insufficient image quality for registration. Failure of the registration to MNI space accounted for most patients excluded. Thus, we included 315 patients in this study. Mean age was 66 (SD ± 16) years, 146 (46.3%) were female, mean NIHSS was 10 (±7, IQR 11, median 8). Mean DWI lesion volume was 22.1 (±35, IQR 24.7, median 7.3) ml. Data on stroke etiology as assessed by the Trial of Org 10172 in Acute Stroke Treatment classification was available for 256 patients [large artery sclerosis: 78 (24.8%), cardioembolism: 104 (33%), small-vessel occlusion: 15 (5.1%), stroke of other determined cause: 24 (7.6%), and stroke of undetermined cause: 34 (10.8%)].

**Figure 1:**
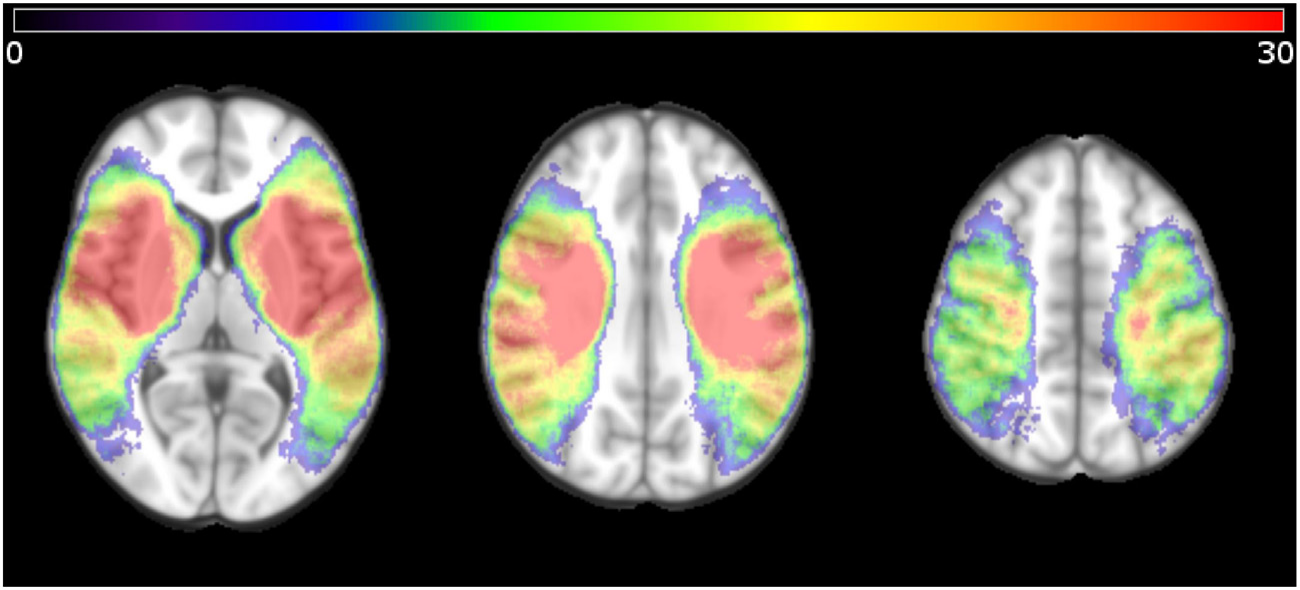
Distribution of ischemic voxels in standard Montreal Neurologic Institute space for all patients studied, displayed as a heat map indicating the number of patients with ischemia in the respective voxel. Only patients with unilateral lesions were included in the study.

Median ASPECTS by visual scoring was 9 (range 1–10), 84 (26.7%) patients had ASPECTS ≤7, 31 (9.8%) patients ASPECTS ≤5. The most frequently affected region on visual scoring was the insular ribbon (I) being affected in 53.7%, M4 was least frequently affected (7.6%). Table 1 shows the frequency of lesions for each individual ASPECTS region. Figure 1 illustrates infarct distribution among the 315 patients included in our analysis.

**Table 1:** Absolute numbers and percentage of all 315 patients with lesions in the respective Alberta Stroke Program Early Computed Tomography Score (ASPECTS) region using visual and automated scoring approaches.

Method	Region									
	Caudate	Internal capsule	Insular ribbon	Lentiform	M1	M2	M3	M4	M5	M6
Visual, *n* (%)	42 (13.3)	37 (11.7)	169 (53.7)	34 (10.8)	26 (8.3)	70 (22.2)	60 (19.0)	24 (7.6)	124 (39.4)	51 (16.2)
Automated >0%, *n* (%)	193 (61.3)	267 (84.7)	256 (81.2)	243 (77.1)	262 (83.2)	264 (83.8)	181 (57.5)	278 (88.3)	304 (96.5)	261 (82.9)
Automated >1%, *n* (%)	115 (36.5)	206 (65.4)	200 (63.5)	183 (58.1)	152 (48.3)	127 (40.3)	58 (18.4)	182 (57.8)	238 (75.6)	104 (33)
Automated >10%, *n* (%)	80 (25.4)	107 (34)	155 (49.2)	101 (32.1)	70 (22.2)	61 (19.4)	16 (5.1)	58 (18.4)	96 (30.5)	29 (9.2)
Automated >20%, *n* (%)	63 (20)	63 (20)	117 (37.1)	74 (23.5)	42 (13.3)	44 (14)	4 (1.3)	23 (7.3)	52 (16.5)	14 (4.4)

The combined volume of all regions of the probabilistic ASPECTS template in MNI space 371 ml. Volumes for subcortical regions were considerably smaller (C: 2.8 ml, IC: 6.7 ml, I: 13.6 ml, and L: 4.8 ml) than for cortical regions (M1: 83.6 ml, M2: 53.4 ml, M3: 52.5 ml, M4: 54.8 ml, M5: 73.9 ml, and M6: 53.4 ml).

**Figure 2:**
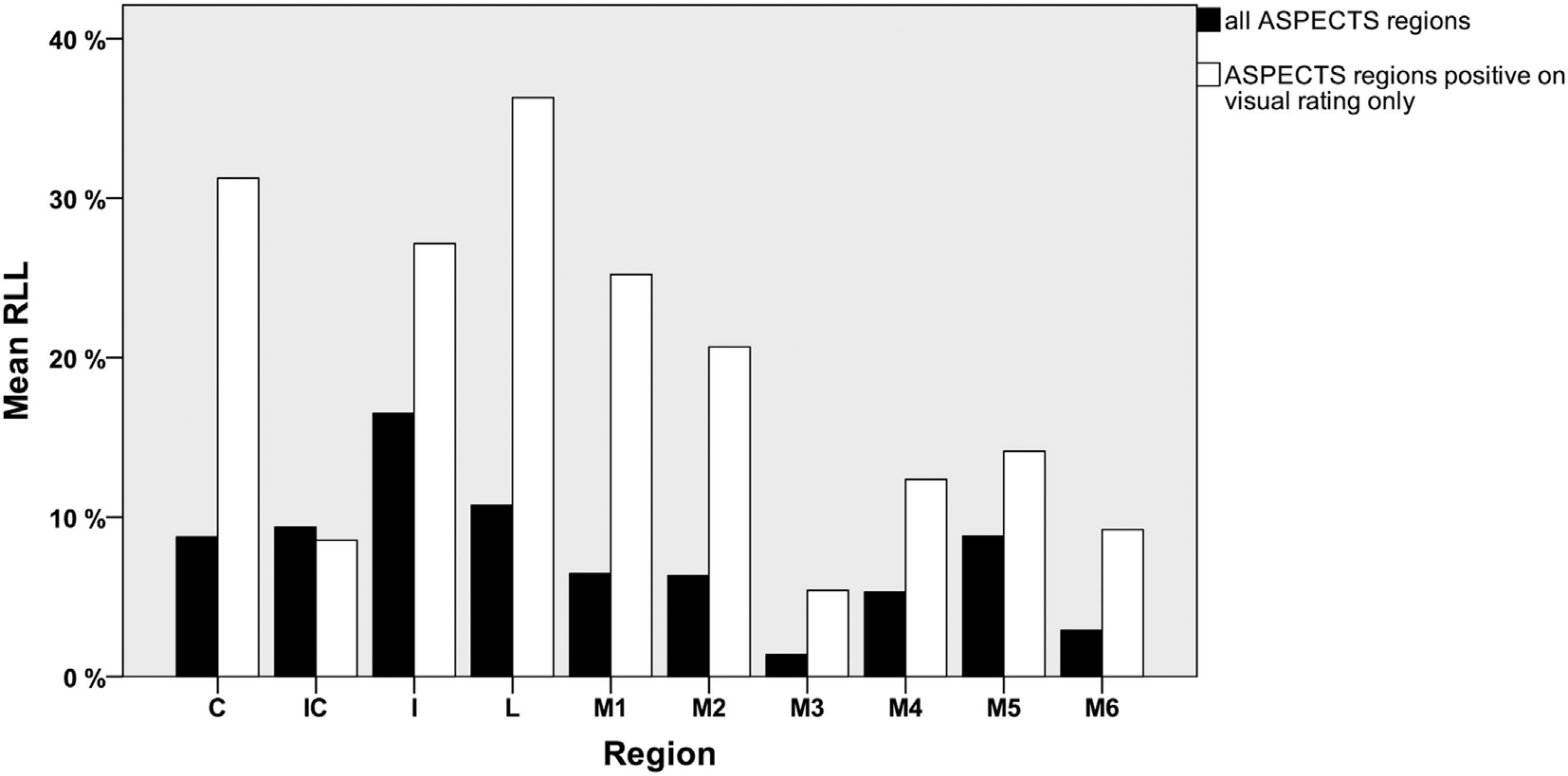
Mean relative lesion load (RLL) *per* ASPECTS region for all patients (black) and mean RLL for visually positive regions only (white).

Figure 2 displays the mean RLL for all regions, and separately shown the mean RLL for regions labeled positive by visual scoring only.

The mean RLL for all ASPECTS regions was 7.7 ± 12.6%, median RLL was 0.9% (IQR 9.634%). For the subcortical regions (C, IC, I, and L), RLL was significantly higher than for the cortical regions (M1–M6): mean 11.4 ± 15.6 vs. 5.2 ± 9.5%, median 2.15 (IQR 20.2%) vs. 0.06% (IQR 5.6%) (*p* < 0.0001). The mean RLL for all regions rated visually positive was 19.5 ± 16.9%, median RLL for all regions rated visually positive was 15.4% (IQR 31.6%). Again subcortical regions had significantly higher overlap than cortical regions [mean 26.4 ± 17.7 vs. 13.9 ± 14%, median 28 (IQR 35.1%) vs. 8.7% (IQR 22.3%) *p* < 0.0001].

**Figure 3:**
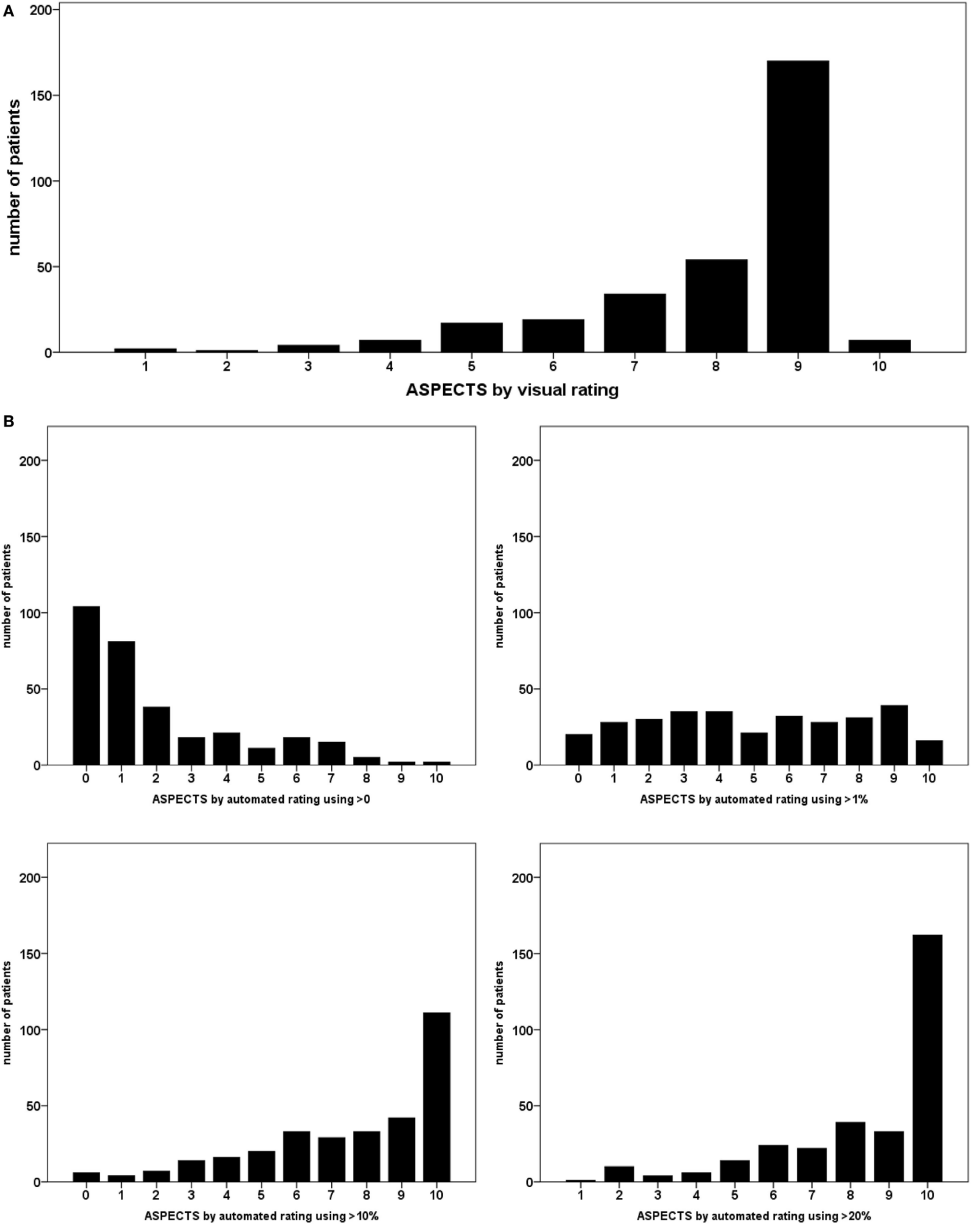
Distribution of Alberta Stroke Program Early Computed Tomography scores over the different relative lesion load (RLL) thresholds in automated rating; each bar represents the number of patients allotted the respective overall score; **(A)** visual rating; **(B)** automated rating using RLL thresholds.

Table 1 lists the number and percentage of patients with lesions per ASPECTS region for the different thresholds. Figure 3 illustrates the distribution of overall ASPECTS scores for all rating approaches. Table 2 lists descriptive statistics for the distribution of ASPECTS values for the different analysis methods. Additionally, the number of patients below the established exclusion thresholds ≤7 and ≤5 for each rating strategy are listed in Table 2.

**Table 2:** Median, minimum, maximum, interquartile range of Alberta Stroke Program Early Computed Tomography Score, number of patients below ≤7, and ≤5 thresholds values using different rating methods.

Method	Median	Minimum	Maximum	IQR	≤7, *n*(%)	≤5, *n*(%)
Visual	9	1	10	7–9	84 (26.7)	31 (9.8)
>0	1	0	10	0–3	306 (97.1)	273 (86.7)
>1%	5	0	10	3–8	229 (72.7)	169 (53.7)
>10%	8	0	10	6–10	129 (41.0)	67 (21.3)
>20%	10	1	10	7–10	81 (25.7)	35 (11.1)

Agreement between visual scoring and the automated approach for calculation of total ASPECTS was low for all thresholds applied with κ_w_ ranging between 0.020 and 0.386. The agreement κ for the dichotomized score (≤7, ≤5) was better, ranging up to moderate or even substantial for >10 or >20% thresholds. Agreement was different for individual ASPECTS regions with poorest agreement for the internal capsule (IC) (mean agreement across all thresholds 0.005) and best agreement for the insula (I) (0.53) and the central MCA region (M2) (0.424). For a detailed list of κ and κ_w_ values over all ASPECTS regions and for thresholds used see Table 3.

**Table 3:** Agreement between visual and automated scoring for total Alberta Stroke Program Early Computed Tomography Score (ASPECTS) (κ_w_), dichotomized for ≤7, and ≤5 thresholds and each ASPECTS region (κ).

	Visual and automated >0%	Visual and automated >1%	Visual and automated >10%	Visual and automated >20%	Mean agreement
Overall score	0.020	0.151	0.386	0.381	
Dichotomized ≤7	0.020	0.219	0.480	0.450	
Dichotomized ≤5	0.033	0.148	0.528	0.695	
Caudate	0.166	0.296	0.424	0.490	0.344
Internal capsule	0.030	0.070	0.024	−0.103	0.005
Insular ribbon	0.355	0.605	0.632	0.526	0.53
Lentiform	0.069	0.161	0.373	0.478	0.27
M1	0.036	0.137	0.384	0.476	0.258
M2	0.105	0.452	0.605	0.534	0.424
M3	0.297	0.500	0.313	0.072	0.296
M4	0.022	0.069	0.153	0.149	0.098
M5	0.035	0.141	0.308	0.260	0.186
M6	0.077	0.415	0.264	0.223	0.245

*For individual ASPECTS region the mean agreement over all thresholds are shown*.

There were significant negative correlations between ASPECTS and NIHSS for visual rating (*r* = −0.46, *p* < 0.0001) and automated rating for thresholds applied (>0: *r* = −0.59, *p* < 0.0001; ≥1%: *r* = −0.62, *p* < 0.0001; ≥10%: *r* = −0.62, *p* < 0.0001; ≥20%: *r* = −0.62, *p* < 0.0001).

**Figure 4:**
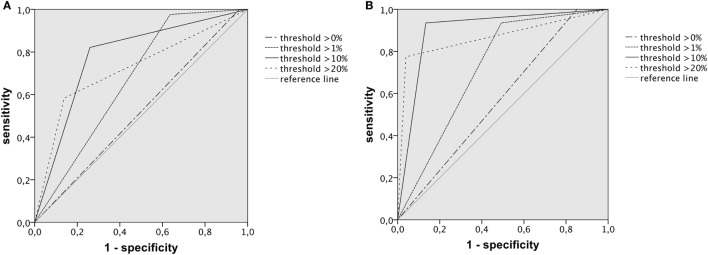
ROC curves illustrating the performance of dichotomized Alberta Stroke Program Early Computed Tomography Score (ASPECTS) determined by automated rating approaches for prediction of the respective visual cut-off; **(A)** for ASPECTS ≤7, AUC was highest for the >10% relative lesion load (RLL) threshold with 0.781, 95% confidence interval of 0.723–0.839; **(B)** for ASPECTS ≤5, AUC was highest for the 10% RLL threshold with 0.901, 95% confidence interval of 0.845–0.956.

ROC curves for dichotomized visual ASPECTS ≤7 and ≤5 and the threshold-based methods are shown in Figure 4.

## Discussion

Alberta Stroke Program Early Computed Tomography Score is a widely used tool for assessment of acute stroke lesions on non-contrast or perfusion CT and DWI-MRI. Nevertheless, the usual visual method of rating is ultimately subjective and open questions remain, especially when MRI is used. For application to DWI lesions, clear instructions on how to calculate the score are lacking. We compared an automated quantitative analysis based on the overlap of the individual lesion mask and a probabilistic ASPECTS template with the usual visual analysis.

As a main result, we found low overall agreement between visual ASPECTS scoring and automated scoring no matter which threshold was used. We could not identify an RLL threshold that perfectly matches the visual rating. The average RLL of all visually positive regions was 19.5%. Still even the higher thresholds tested (>10 and >20%) yielded differing results for the individual regions with only fair overall agreement.

The different thresholds of automated ASPECTS computation resulted in largely different numbers of patients below the ASPECTS cut-offs that have been suggested to exclude patients from endovascular treatment, i.e., ASPECTS ≤7 or ≤5 (5, 7).

For the higher RLL thresholds (>10 and >20%) the dichotomized scoring showed at least moderate agreement with visual scoring with regards to ≤7 or ≤5 ASPECTS cut-offs. Thus, automated scoring with higher RLL thresholds appears to be close to visual rating, at least when aiming at dichotomized classification according to ASPECTS. However, our findings highlight the relevance of lesion load thresholds as they have a critical impact on patient selection by ASPECTS.

For the regions labeled affected by visual rating, mean RLL per ASPECTS region varied considerably. Mean RLL of subcortical regions was clearly higher than that of cortical regions. This may to a certain amount reflect the distribution of acute MCA infarction with highest frequency in the basal ganglia, IC, and insula. On the other hand, this imbalance matches with earlier findings describing an imbalance between cortical and subcortical ASPECTS regions (10). Visual rating and automated assessment agreement was worst for IC and superior cortical regions (M4, M5) similar to earlier findings (17). The disparity in agreement between regions further corroborates the hypothesis that ASPECTS regions are unequally weighed, i.e., the same overall score does not necessarily indicate the same lesion load.

Of note, assessment of overall interrater agreement for visual rating of dichotomized ASPECTS on CT and MRI has provided varying results (1, 14, 17–19). In patient with large vessel occlusion, recent studies described slight to moderate agreement for NCCT ASPECTS (20) and slight agreement for DWI-ASPECTS (21) when raters from multiple specialties were evaluated.

It is unclear what kind of implicit threshold is applied for visual rating in every day clinical practice. There appears to be a common mechanism which might include implicit application of higher thresholds for certain regions, e.g., subcortical. There have been other attempts to establish an automated ASPECTS rating (22), which demonstrated a higher sensitivity of the automated approach compared to expert reading. This is at least partially in line with our findings of overall lower scores when using low RLL thresholds. Depending on the threshold applied less lesioned tissue per ASPECTS region was required for a “positive” evaluation on automated compared to visual rating. This could explain a higher sensitivity of automated approaches. Furthermore, software solutions may be suitable to overcome limitations stemming from low interrater reliability.

Overall visual rating seems to be an easily applicable, albeit blunt instrument compared to the meticulous voxel-wise automated approach, which in this case was only partially successful in replicating the visual assessment.

## Limitations

There are considerations that limit generalizability of our findings. Some of the issues mentioned above may be inherent to DWI due to its higher sensitivity for even small lesions and thus may not be applicable for ASPECTS on CT. However, an unequal weighing of different ASPECTS regions would also affect scoring on non-contrast CT or CT perfusion imaging (10).

The relatively low mean lesion volume in the sample studied may represent a further limitation to our study. Only a minority of the patients analyzed here presented with large artery occlusion; the sample may thus differ from a population in whom ASPECTS is applied to weigh benefit and risk of endovascular stroke treatment. To better reflect clinical reality, our results require confirmation in a sample of patients with emergent large artery occlusion only.

## Conclusion

The results of our study add new insights to the research describing characteristics and limitations of the use of ASPECTS to evaluate acute stroke imaging. ASPECTS is clearly helpful to standardize assessment of infarct core in acute stroke treatment, for patient selection in clinical trials, as well as for guiding treatment decisions in clinical practice. Nevertheless, one should keep in mind the limitations of the scale, such as the unequal weighing of the different regions and the lack of a formal rule as to the when a region should be considered affected. This gets even more important when ASPECTS is used with imaging modalities more sensitive to acute ischemia than non-contrast CT, e.g., DWI or perfusion CT. Our results highlight the relevance of lesion load thresholds as they have a critical impact on the number of patients excluded by ASPECTS cut-offs. The lack of patients with large artery occlusion and low mean lesion volume in this cohort represent relevant limitations.

Finally, these limitations may be of clinical relevance when ASPECTS cut-off values are used to exclude patients from clinical trials or treatment in clinical practice, e.g., mechanical thrombectomy. The REVASCAT trial already attempted to account for this by defining different ASPECTS cut-offs for CT and DWI (6). Further clarification of rating procedures may be necessary to facilitate reliable transfer of cut-offs between different imaging modalities and rating strategies.

## Ethics Statement

This study was carried out in accordance with the recommendations of Ethik-Kommission der Ärztekammer Hamburg, Germany. The protocol was approved by the Ethik-Kommission der Ärztekammer Hamburg. All subjects gave written informed consent in accordance with the Declaration of Helsinki. PRE-FLAIR was conducted by an international consortium of researchers within the Stroke Imaging Repository (STIR) and Virtual International Stroke Trials Archive (VISTA) Imaging research groups. PRE-FLAIR included individual datasets from eight participating stroke centers and two studies. The study was approved by the local ethics committees at all centers. Either written or verbal informed consent was obtained for all patients as required by local legislation.

## Author Contributions

JS, BC, CM, ME, MK, OW, D-WK, DL, TT, OS, BC, ML, SW, JF, AK, JBF, CG, and GT all contributed to data collection, drafting, and revising of the manuscript. JS, BC, CM, GT, and AK conducted data analysis.

## Conflict of Interest Statement

JBF has received fees as a board member, consultant, or lecturer from Boehringer Ingelheim, Lundbeck, Siemens, Sygnis, and Synarc. CG has received fees as a consultant or lecture fees from Bayer Vital, Boehringer Ingelheim, EBS technologies, Glaxo Smith Kline, Lundbeck, Pfizer, Sanofi Aventis, Silk Road Medical, and UCB. DL has received consultant fees for Imaging Core Laboratory for Stryker and Medtronic. TT has received a national grant from the French Government (PHRC). OW was supported in part by grants from the National Institutes of Health (R01NS059775, P50NS051343, R01NS063925), received consulting fees from Penumbra Inc., and received royalties from General Electric, Olea, and Imaging Biometrics. GT, BC, BC, CM, AK, SC, ME, JF, D-WK, MK, ML, JS, OS, and SW have no conflicts of interest to report.
