# Supplementary material for: Impact of Lesion Load Thresholds on Alberta Stroke Program Early Computed Tomographic Score in Diffusion-Weighted Imaging
Source: Front Neurol. 2018 Apr 23;9:273. doi: 10.3389/fneur.2018.00273 (PMC5926541; doi:10.3389/fneur.2018.00273)

Supplementary Material

**Impact of lesion load thresholds on Alberta Stroke Program Early Computed Tomographic Score in diffusion-weighted imaging**

Julian Schröder^1*^, MD; Bastian Cheng^1^, MD; Caroline Malherbe^1,14^, PhD; Martin Ebinger^2,2b^, MD; Martin Köhrmann^3^, MD; Ona Wu^4^, PhD; Dong-Wha Kang^5^, MD; David S Liebeskind^6^, MD; Thomas Tourdias^7^, MD; Oliver C Singer^8^, MD; Bruce Campbell, MD^9^; Marie Luby^10^, PhD; Steven Warach^11^, MD; Jens Fiehler^12^, MD; André Kemmling^13^, MD; Jochen B Fiebach^2^, MD; Christian Gerloff^1^, MD; Götz Thomalla^1^, MD; on behalf of the STIR and VISTA Imaging Investigators

^1^ Klinik und Poliklinik für Neurologie, Kopf- und Neurozentrum, Universitätsklinikum Hamburg-Eppendorf, Martinistr. 52, 20246 Hamburg, Germany
^2^ Centrum für Schlaganfallforschung Berlin, Charité-Universitätsmedizin Berlin, Charitéplatz 1, 10117 Berlin, Germany
^2b^ Klinik für Neurologie, Charité - Universitätsmedizin Berlin, Charitéplatz 1, 10117 Berlin, Germany
^3^ Klinik für Neurologie, Universität Erlangen-Nürnberg, Maximiliansplatz 2, 91054 Erlangen, Germany
^4^ Athinoula A Martinos Center for Biomedical Imaging, Department of Radiology, Massachusetts General Hospital, Harvard Medical School, 25 Shattuck Street Boston, MA02115, USA
^5^ Department of Neurology, Asan Medical Center, University of Ulsan College of Medicine, 388-1 Pungnap-dong, Songpa-ku, Seoul 138-736, South Korea
^6^ Department of Neurology, University of California, Los Angeles, 710 Westwood Plaza, Los Angeles, CA 90095, USA
^7^Univ. de Bordeaux, CHU de Bordeaux, Service de NeuroImagerie Diagnostique de Thérapeutique, Place Amélie Raba Léon, F-33000 Bordeaux, France
^8^ Klinik für Neurologie, Universitätsklinikum Frankfurt, Schleusenweg 2-16, 60528 Frankfurt
^9^ Department of Medicine and Neurology, Melbourne Brain Centre at the Royal Melbourne Hospital, University of Melbourne, Parkville, Australia
^10^ National Institute of Neurological Disorders and Stroke (NINDS), National Institutes of Health (NIH), Bethesda, MD, USA
^11^ Department of Neurology, Dell Medical School, University of Texas at Austin, Austin, TX, USA
^12^ Klinik und Poliklinik für Neuroradiologische Diagnostik und Intervention, Universitätsklinikum Hamburg-Eppendorf, Martinistr. 52, 20246 Hamburg, Germany

^13^ Institut für Neuroradiologie, Universitätsklinikum Schleswig-Holstein, Ratzeburger Allee 160, 23528 Lübeck, Germany

^14^ Institut für Computational Neuroscience, Universitätsklinikum Hamburg-Eppendorf, Martinistr. 52, 20246 Hamburg, Germany

***Corresponding author:**
E-Mail: jul.schroeder@uke.de (JS)

**Supplementary Figures:** Details of probabilistic ASPECTS template. ASPECTS regions were manually delineated on 221 normal NCCT scans and registered to standard space. A probabilistic template was then generated from the registered images. The percentage of infarcted volume within a region is calculated weighted by anatomical probability.
From Kemmling et al. Clinical outcome prediction by quantitative regional infarction of ASPECTS regions in stroke**,** Clinical Neuroradiology, 24(Supplement 1):47, 2014.

- ASPECTS regions were manually segmented on 221 CT images by two raters using all slices covering the entire MCA territory
- Binary ASPECTS maps based on each exam were then affine registered to standard MNI space


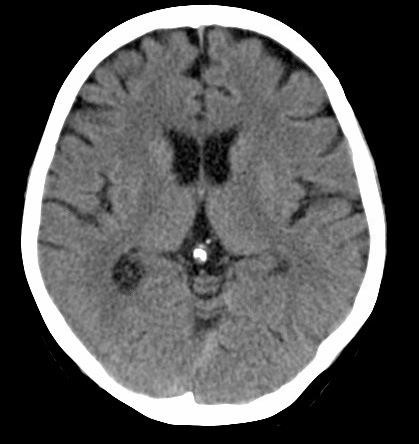

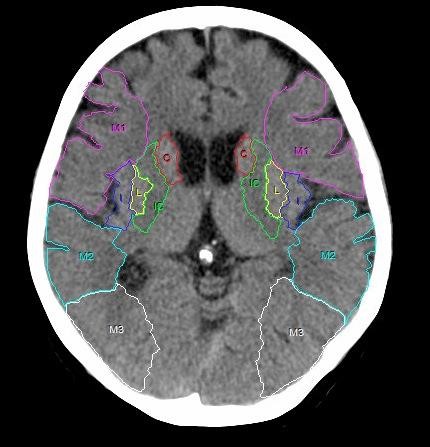

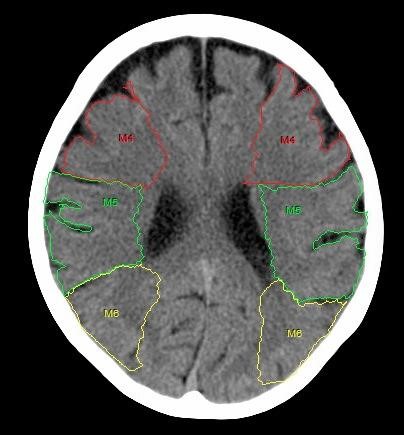

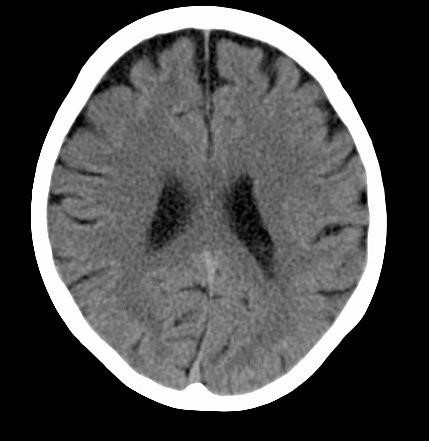

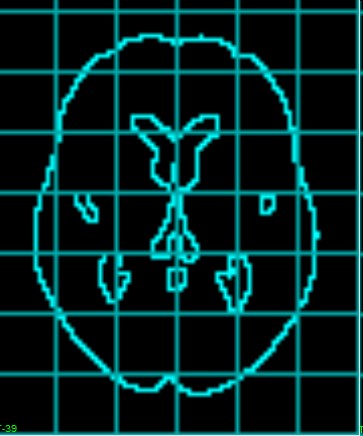

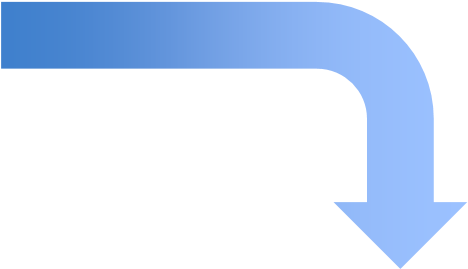


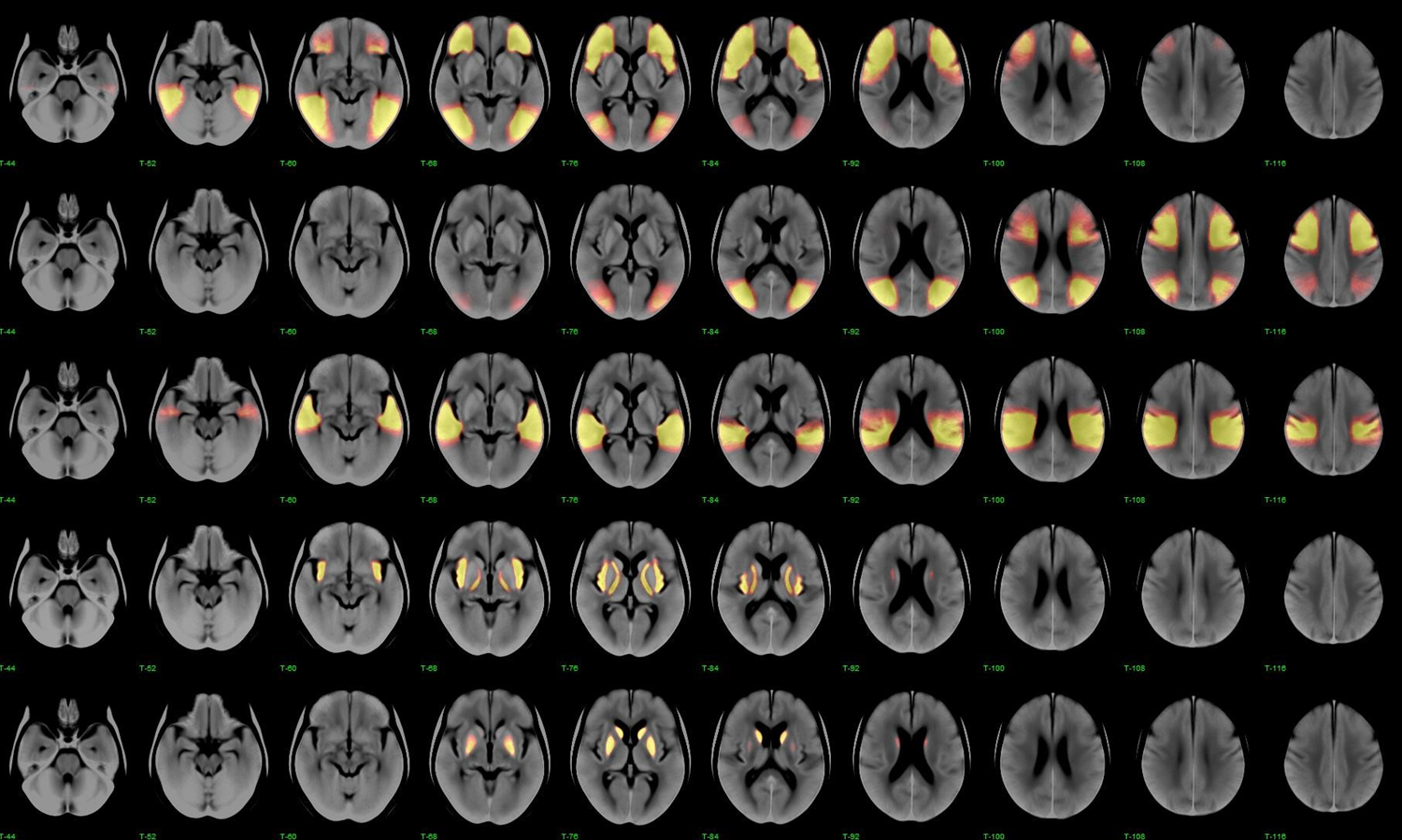


**I, IC**

**C, L**

**M2, M5**

**M4, M6**

**M1, M3**


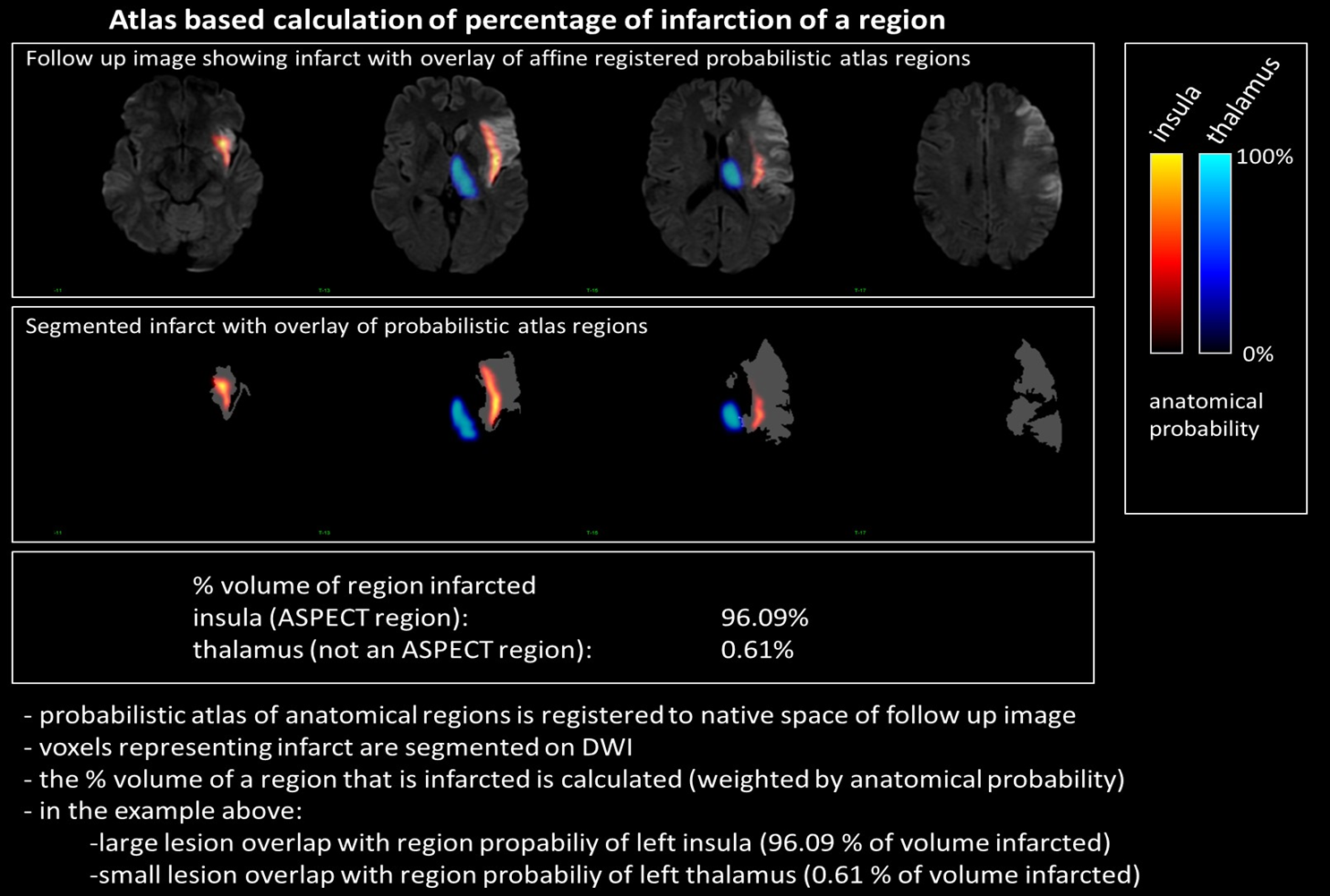

Supplement: Supplementary file 1 [file data_sheet_1.docx]
